# Jejunal perforation in gallstone ileus – a case series

**DOI:** 10.1186/1752-1947-1-157

**Published:** 2007-11-28

**Authors:** Louise E Browning, Jeremy D Taylor, Sue K Clark, Nariman D Karanjia

**Affiliations:** 1 Department of Surgery, University Hospital Lewisham, Lewisham, UK; 2 Department of Radiology, St. George's Hospital, London, UK; 3 Department of Surgery, St Mark's Hospital, Harrow, UK; 4 Department of Surgery, Royal Surrey County Hospital, Surrey, UK

## Abstract

**Introduction:**

Gallstone ileus is an uncommon complication of cholelithiasis but an established cause of mechanical bowel obstruction in the elderly. Perforation of the small intestine proximal to the obstructing gallstone is rare, and only a handful of cases have been reported. We present two cases of perforation of the jejunum in gallstone ileus, and remarkably in one case, the gallstone ileus caused perforation of a jejunal diverticulum and is to the best of our knowledge the first such case to be described.

**Case presentations:**

Case 1

A 69 year old man presented with two days of vomiting and central abdominal pain. He underwent laparotomy for small bowel obstruction and was found to have a gallstone obstructing the mid-ileum. There was a 2 mm perforation in the anti-mesenteric border of the dilated proximal jejunum. The gallstone was removed and the perforated segment of jejunum was resected.

Case 2

A 68 year old man presented with a four day history of vomiting and central abdominal pain. Chest and abdominal radiography were unremarkable however a subsequent CT scan of the abdomen showed aerobilia. At laparotomy his distal ileum was found to be obstructed by an impacted gallstone and there was a perforated diverticulum on the mesenteric surface of the mid-jejunum. An enterolithotomy and resection of the perforated small bowel was performed.

**Conclusion:**

Gallstone ileus remains a diagnostic challenge despite advances in imaging techniques, and pre-operative diagnosis is often delayed. Partly due to the elderly population it affects, gallstone ileus continues to have both high morbidity and mortality rates. On reviewing the literature, the most appropriate surgical intervention remains unclear.

Jejunal perforation in gallstone ileus is extremely rare. The cases described illustrate two quite different causes of perforation complicating gallstone ileus. In the first case, perforation was probably due to pressure necrosis caused by the gallstone. The second case was complicated by the presence of a perforated jejunal diverticulum, which was likely to have been secondary to the increased intra-luminal pressure proximal to the obstructing gallstone.

These cases should raise awareness of the complications associated with both gallstone ileus, and small bowel diverticula.

## Introduction

Gallstone ileus is an uncommon surgical emergency that occurs almost exclusively in the elderly, with a peak incidence between 65 and 75 years of age. However, it is of increasing significance with the current demographic shift towards an elderly population.

Perforation of the small intestine proximal to the obstructing gallstone is rare with less than 10 cases ever having been described. We present two cases of perforation of the jejunum in gallstone ileus, and remarkably in one case, the gallstone ileus caused perforation of a jejunal diverticulum and is to the best of our knowledge the first such case to be described.

The main objective of this review is to critically evaluate the known difficulties associated with the diagnosis and treatment of gallstone ileus, and report a rare complication, thus increasing the awareness of jejunal perforation and small bowel diverticula.

## Case presentation

### Case 1

A 69 year old man presented with two days of vomiting and central abdominal pain. He suffered with hypertension and gastro-oesphageal reflux disease but had never undergone surgery. His bilirubin was 28 μmmoll^-1^, otherwise liver function tests were normal. An abdominal radiograph showed dilated loops of small bowel. He underwent a laparotomy at which a gallstone was found obstructing the mid-ileum. There was a 2 mm perforation in the anti-mesenteric border of the dilated proximal jejunum. The gallstone was removed via enterolithotomy and the perforated segment of jejunum was resected. He made an uneventful recovery.

### Case 2

**Figure 1:**
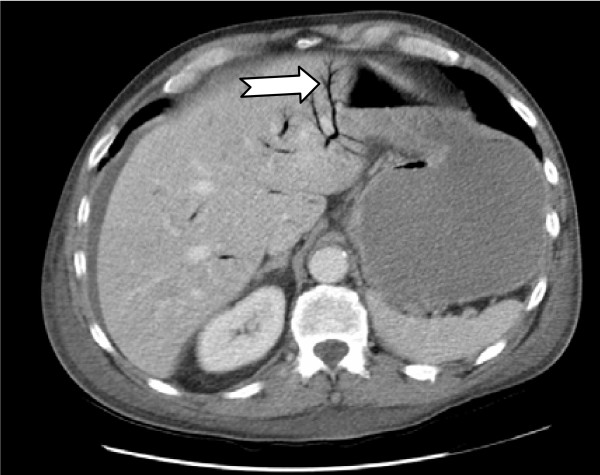
CT scan showing aerobilia (arrow) consistent with a cholecysto-enteric fistula and free fluid within the abdomen.

**Figure 2:**
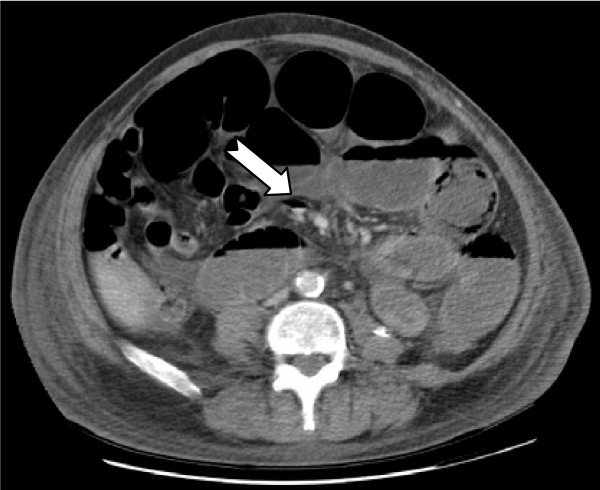
CT scan showing multiple dilated loops of small bowel with free fluid and air seen on the mesenteric border of the mid jejunum (arrow) suggesting perforation of the small bowel.

A 68 year old man presented with a four day history of vomiting and central abdominal pain. He was hypertensive and had no history of previous abdominal surgery. Chest and abdominal radiography were unremarkable. A subsequent CT scan of the abdomen showed aerobilia (Figure 1) and small bowel dilatation to the distal ileum with accompanying free intra-abdominal fluid (Figure 2). At laparotomy his distal ileum was found to be obstructed by an impacted gallstone and there was a perforated diverticulum on the mesenteric surface of the mid-jejunum. In retrospect, the CT also showed a small pocket of air within the mesentery and later these findings were later confirmed on histology. The gallstone was removed via enterolithotomy and the perforated segment of jejunum was resected. He made an uneventful recovery.

## Discussion

In both of the above cases, the patients presented with small bowel obstruction with a preceding history of several days of abdominal pain and vomiting. This protracted history is the classical presentation of gallstone ileus, with the majority of patients suffering with abdominal pain and vomiting for at least three days prior to presentation [1]. This is caused by the gallstone moving down the intestine and intermittently obstructing before becoming impacted, so called 'tumbling obstruction'.

Apart from small bowel obstruction, the abdominal radiographs showed none of the three radiological signs of gallstone ileus, namely aerobilia, aberrantly located gallstone or a change in location of a previous gallstone. This is often the case, as two from these three signs are present in only 40–50 per cent of patients with gallstone ileus. It is well recognized that gallstone ileus is a difficult clinical and radiological diagnosis [2] and only a decade ago, correct pre-operative diagnosis was as low as 20%. However, recent advances in ultrasonography and computerised tomography can show the presence and location of gallstones, fistulae and aerobilia. Unreserved use of these imaging techniques, in combination with plain abdominal radiographs, can expedite the correct diagnosis in over 50% of cases and decrease preoperative delay [1].

The mainstay of surgical treatment for gallstone ileus is prompt relief of the small bowel obstruction by removing the gallstone by open enterolithotomy. However, to date, controversy reigns over both the most appropriate approach, and the proper extent of surgery. There are two well recognised surgical procedures; the one stage procedure combines enterolithotomy, cholecystectomy and fistula repair, whereas the two stage procedure consists of enterolithotomy alone and biliary surgery at a later stage if indicated. Whether it is preferable to perform the more complex one stage operation, or the simpler enterolithotomy, continues to be actively debated.

Support for enterolithotomy alone, results from it being the minimalist surgery possible in order to relieve bowel obstruction in the emergency situation. It is safe in both low and high-risk patients, requires a shorter operating time than the one stage procedure, and is technically less demanding [3]. It can be combined with an elective laparoscopic cholecystectomy at a later date if biliary symptoms persist, but in most cases enterolithotomy alone is adequate treatment in the elderly patient and subsequent cholecystectomy is not mandatory [2].

It is argued that the one stage procedure significantly decreases mortality because removing the gallbladder and biliary-enteric fistula prevents future recurrence of gallstone ileus, and recurrent biliary symptoms with their associated morbidity and mortality [4]. It also obviates the need for a second operation.

The largest review to date, of 1001 reported case of gallstone ileus found the one-stage procedure carried an associated mortality of 16.9%, compared to 11.7% for simple enterolithotomy. Also, interestingly the recurrence rate of gallstone ileus was less than 5 per cent, and only 10 per cent of patients required re-operation for continued symptoms related to the biliary tract [5]. A more recent study reported similar mortality rates, and concluded that urgent fistula repair is associated with a high rate of complications having found the morbidity rate for the one stage group to be twice that of enterolithotomy alone [3].

Despite great advances in peri-operative care over the past few years, mortality rates for gallstone ileus remain high, in the region of 15 – 18% [3-5]. This is partly due to the elderly patient population having multiple medical co-morbidities and one study showed 86% to have an ASA grade of 3 or 4 at the time of surgery. This subset of patients could potentially benefit from a minimally-invasive technique. Thus, several laparoscopic approaches have been reported including laparoscopic-assisted enterolithotomy [6], laparoscopic enterolithotomy alone, and in combination with staged laparoscopic cholecystectomy and fistula closure. In the laparoscopic-assisted procedure, diagnostic laparoscopy is used to identify the exact location of the gallstone in the small bowel. A small, targeted incision can then be made directly over the stone and routine enterolithotomy performed.

Other than the obvious benefits of expedious discharge, it offers the opportunity of diagnostic laparoscopy alone and the chance to perform the enterolithotomy laparoscopically, when clinically appropriate and the expertise is available [7]. In several reported cases, diagnostic laparoscopy has presented the opportunity for simple disimpaction of the gallstone into the large bowel [8]. As with open surgery, controversy exists as to the indication of timing and surgical approach to laparoscopic cholecystectomy and fistula repair.

In both of the presented cases, at the time of surgery the patients were both high risk (ASA 3) and required the minimalist surgery possible. Simple enterolithotomy alone was performed to remove the gallstone, combined with mandatory small bowel resection to excise the perforated jejunum. Diagnostic laparoscopy would have been possible in both cases and indeed would have aided the diagnosis and allowed for a targeted incision. Of note however, is although the jejunal perforation in the first case may have been easily visible, the jejunal diverticular perforation was between the mesenteric folds and may have been concealed.

Perforation of the jejunum in gallstone ileus is very rare indeed. A review of 458 cases of gallstone ileus reported only two cases of jejunal perforation [9]. The perforation occurs either at the site of impaction, or at previous sites of obstruction, as the gallstone tumbles down the intestine, and is thought to be the result of the gallstone causing pressure necrosis of the jejunal wall. This is the likely cause of the anti-mesenteric jejunal perforation described in the first case.

Jejunal diverticula have a prevalence of approximately 1% in the general population and account for 80% of all small bowel diverticula. Of note, they affect a similar age group to gallstone ileus as the prevalence increases with age and peaks at the six and seventh decades [10]. Jejunal diverticula are *acquired *and thought to be pulsation lesions or 'false' diverticula in contrast to 'true' *congenital *diverticula, such as, Meckelian diverticula. They arise from the mesenteric border of the bowel and are formed by the herniation of mucosa and submucosa through the muscular layer at a point of weakness where arteries enter the bowel wall. Fortunately, most remain asymptomatic and the diagnosis is frequently made incidentally by radiological investigation or at laparotomy. The reported complications of jejunal diverticula include perforation, inflammation, abscess formation and intestinal obstruction, and occur in approximately 6–10% of patients. Perforation is less common than that of large bowel diverticula perhaps, because the intra-luminal pressure is less.

In the second of our cases we believe that the presence of a gallstone obstructing the lumen of the ileum caused a rise in the intra-luminal pressure of the proximal jejunum and was responsible for the perforation of a diverticulum arising from the mesenteric aspect. At operation the diagnosis may be difficult as most diverticula form between the two folds of mesentery, resulting more often in a mesenteric abscess rather than free perforation.

## Conclusion

Gallstone ileus accounts for one to four per cent of mechanical intestinal obstruction and particularly occurs in the 65 to 75 year age group. However, perforation of the small intestine proximal to the obstructing gallstone is very rare. The cases described illustrate two quite different causes of perforation complicating gallstone ileus, and highlights the difficulties associated with pre-operative diagnosis and subsequent management. The cases should also raise awareness of small bowel diverticula.

## Competing interests

The author(s) declare that they have no competing interests.

## Authors' contributions

LB identified the relevant cases, conducted the literature search and wrote the discussion

JT wrote the case presentation and prepared the figures

SC and NK were involved in conception of the article and revising it critically for important intellectual data before final approval

All authors read and approved the final manuscript

## Consent

Written informed consent was obtained from both patients for publication of this case report and the accompanying images
